# Serotonergic system in vivo with [^11^C]DASB PET scans in GTP-cyclohydrolase deficient dopa-responsive dystonia patients

**DOI:** 10.1038/s41598-022-10067-5

**Published:** 2022-04-15

**Authors:** Elze R. Timmers, Débora E. Peretti, Marenka Smit, Bauke M. de Jong, Rudi A. J. O. Dierckx, Anouk Kuiper, Tom J. de Koning, David Vállez García, Marina A. J. Tijssen

**Affiliations:** 1 Department of Neurology, University Medical Center Groningen, University of Groningen, PO Box 30.001, 9700 RB Groningen, The Netherlands; 2 Expertise Center Movement Disorders Groningen, University Medical Center Groningen (UMCG), PO Box 30.001, 9700 RB Groningen, The Netherlands; 3 Department of Nuclear Medicine and Molecular Imaging, University Medical Center Groningen (UMCG), University of Groningen, PO Box 30.001, 9700 RB Groningen, The Netherlands; 4 Department of Clinical Sciences, Lund University, Box 117, 221 00 Lund, Sweden

**Keywords:** Diseases, Medical research, Neurology, Pathogenesis

## Abstract

GTP-cyclohydrolase deficiency in dopa-responsive dystonia (DRD) patients impairs the biosynthesis of dopamine, but also of serotonin. The high prevalence of non-motor symptoms suggests involvement of the serotonergic pathway. Our study aimed to investigate the serotonergic system in vivo in the brain of`DRD patients and correlate this to (non-)motor symptoms. Dynamic [^11^C]DASB PET scans, a marker of serotonin transporter availability, were performed. Ten DRD, 14 cervical dystonia patients and 12 controls were included. Univariate- and network-analysis did not show differences in binding between DRD patients compared to controls. Sleep disturbances were correlated with binding in the dorsal raphe nucleus (all participants: r_*s*_ = 0.45, *p* = 0.04; patients: r_s_ = 0.64, *p* = 0.05) and participants with a psychiatric disorder had a lower binding in the hippocampus (all participants: *p* = 0.00; patients: *p* = 0.06). Post-hoc analysis with correction for psychiatric co-morbidity showed a significant difference in binding in the hippocampus between DRD patients and controls (*p* = 0.00). This suggests that psychiatric symptoms might mask the altered serotonergic metabolism in DRD patients, but definite conclusions are difficult as psychiatry is considered part of the phenotype. We hypothesize that an imbalance between different neurotransmitter systems is responsible for the non-motor symptoms, and further research investigating multiple neurotransmitters and psychiatry in DRD is necessary.

## Introduction

Dopa-responsive dystonia (DRD) is a rare inherited metabolic disorder in which patients present with dystonia characterized by diurnal fluctuations and an excellent response to levodopa treatment^1^. The most common type of dopa-responsive dystonia is caused by an autosomal dominant inherited mutation in the *GCH1* gene (OMIM#600225) affecting the enzyme GTP cyclohydrolase 1^2^. This enzyme is the first and rate-limiting step in the biosynthesis of tetrahydrobiopterin (BH4), which is an essential co-factor involved in the biosynthesis of mono-amine neurotransmitters, such as dopamine and serotonin.

The biochemical changes in dopamine are considered to be responsible for the motor phenotype of dystonia and Parkinsonism in DRD patients and hence the excellent response to levodopa therapy. Besides these motor manifestations, attention has been drawn to non-motor symptoms in DRD with an increased prevalence of psychiatric and sleep disorders compared to controls^3–5^. The pathophysiology of non-motor symptoms in dystonia is not known, however, serotonin is suggested to play an important role^1,6^. As already indicated, the biosynthesis of serotonin, identical to that of dopamine, is impaired due to the *GCH1* mutation and subsequent deficiency of BH4. In line with this, decreased levels of 5-Hydroxyindoleacetic acid (5-HIAA), the final breakdown product of serotonin, in cerebral spinal fluid of DRD patients has been described^3,7^. Furthermore, the serotonergic system is important in the regulation of mood and sleep, and is implicated in the pathophysiology of many psychiatric disorders.

To investigate the potential role of the serotonergic system in the brain of DRD patients, positron emission tomography (PET) can be used. The radiotracer [^11^C]-3-amino-4-(2-dimethylaminomethyl-phenylsulfanyl)benzonitrile ([^11^C]DASB) is a well-validated and selective radio-ligand that binds to the serotonin reuptake transporter (SERT) and qualifies as an excellent biomarker to study the serotonergic system in vivo^8^.

Dystonia, including DRD, is considered to be a network disorder, with multiple brain regions involved in its pathophysiology^9^. Therefore, a network-based analysis may provide useful additional information. A frequently used method to create disease-specific patterns is the scaled subprofile model, which is a spatial covariance method based on principal component analysis (SSM/PCA)^10^. Recently, a disease-specific covariance pattern on [^11^C]DASB PET scans was detected using this method in Parkinson’s disease^11^.

To the best of our knowledge, there are no published studies investigating SERT status in vivo in DRD patients using [^11^C]DASB PET scans. Therefore, this study aimed to examine group differences in serotonergic system integrity between controls and DRD patients using both univariate- and network-based analysis. The second aim is to investigate the relationship between (non-)motor symptoms and the serotonergic system in the brain of DRD patients.

## Materials and methods

### Participants and clinical measures

Ten DRD patients with a confirmed mutation in the *GCH1* gene were included. Data of two control groups, of which data was published before^12^, were used: (1) 14 clinically diagnosed idiopathic cervical dystonia (CD) patients, (2) 12 controls without a movement disorder. No pilot PET data on SERT receptor availability in patients with dystonia was available, therefore, we based our sample size on previous studies on SERT status in (neuro)psychiatric disorders which used 10–15 participants^13,14^. None of the participants were using serotonergic medication that influences serotonin, including antidepressants, or had relevant neurological comorbidity.

Clinical and demographic characteristics of the patients were obtained through a standardized interview. The severity of dystonia was assessed with the Burke Fahn Marsden Dystonia Rating Scale (BFMDRS) and the Clinical Global Impression Scale (CGI). Non-motor symptoms were assessed using validated questionnaires. The Mini International Neuropsychiatric Interview—PLUS (MINI-PLUS) was used to evaluate the presence of psychiatric disorders. Severity of depression, anxiety, fatigue, excessive daytime sleepiness and quality of sleep were assessed with the Beck Depression Inventory (BDI), the Beck Anxiety Inventory (BAI), the Fatigue Severity Scale (FSS), the Epworth Sleepiness Scale (ESS) and the Pittsburgh Sleep Quality Index (PSQI), respectively.

Written informed consent was obtained from all subjects according to the Declaration of Helsinki. This study was approved by the medical ethical committee of the UMCG (METc 034/14).

### SERT polymorphism

Three different alleles in the SERT-linked polymorphic region (5-HTTLPR) are known to be associated with different transcriptional activity and, therefore, influence the number of expressed SERT^15^. A set of specific single-nucleotide polymorphisms in the 5-HTTLPR was tested as described before^12^.

### Scanning protocol

Individual axial 3D T1-weighted gradient-echo images (3T Intera, Philips, The Netherlands) of the brain were acquired from all participants. PET imaging of DRD patients was performed using the same protocol as previously published for CD patients and controls with either a Biograph 40-mCT or 64-mCT (Siemens Healthcare, USA). The protocol consisted of a 60-min dynamic acquisition scan starting simultaneously with an intravenous bolus of [^11^C]DASB (mean dose 382 ± 41 MBq)^16^. PET images were reconstructed from list-mode data into 23 frames (7 × 10 s, 2 × 30 s, 3 × 1 min, 2 × 2 min, 2 × 3 min, 5 × 5 min, and 2 × 10 min). Head movement was minimized with a head-restraining band. DRD patients were allowed to continue using their levodopa treatment during the day of the scan.

### Image processing and analysis

Reconstruction of the dynamic [^11^C]DASB images was performed using the 3D OSEM algorithm (3 iterations and 24 subsets), point spread function correction, and time-of-flight, resulting in a matrix of 400 × 400 × 111 of isotropic 2-mm voxels, smoothed with 2-mm filter at full width at half maximum (FWHM).

Image processing was performed with PMOD v3.8 software. We applied movement correction (frames 13–23, using 1–12 as reference) before the individual PET image was matched to the individual MRI. A six-tissue probability map was used to estimate grey and white matter volumes and a normalization of the individual MRI onto the Montreal Neurological Institute (MNI) standard space was calculated and applied to the corresponding PET image. Predefined volumes of interest (VOI) based on Hammers atlas were transformed into the individual MRI space^17^. As no differences between the left and right hemispheres were identified, a weighted average, based on the volume of the region, was calculated from the corresponding regions in both hemispheres. VOIs for the dorsal raphe nucleus (DRN) and median raphe nucleus (MRN) were manually defined by selecting the 80% of the voxels with the highest uptake within a sphere according to visual inspection of the PET image, as described previously (subject-based: sDRN and sMRN)^12^. The DRN and MRN were also defined based on their MNI coordinates (atlas-based: aDRN and aMRN)^18^. A Gaussian kernel of 6-mm FWHM was applied before pharmacokinetic modeling was performed with PXMOD tool of PMOD V4.0 software. Non-displaceable binding potential (*BP*_ND_) parametric maps were calculated using the Simplified Reference Tissue Model 2 (SRTM2) with the cerebellum (excluding vermis) as reference region and the striatum as high-binding region for estimation of *k*_2_′^19^.

For the VOI-based analysis, average *BP*_ND_ values per region in individual space were used. For both voxel-based analysis and SSM/PCA, *BP*_ND_ parametric maps were used in MNI space.

### SSM/PCA analysis

SSM/PCA analysis was performed in order to assess whether a disease-specific pattern of *BP*_ND_ could discriminate DRD from CD and controls. SSM/PCA analysis was performed in MATLAB R2018b using in-house software which was validated against the original study where these mathematical principles are extensively reported^20,21^. In brief: *BP*_ND_ data was masked and centered twice, by subtracting subject and region means to obtain a matrix of subject residuals. A covariance matrix was constructed of these residuals and PCA was applied, resulting in principle components (PCs). The PCs that cumulatively account for 50% of the variance were selected. A stepwise forward combination was used to generate the disease patterns and the pattern with the lowest Akaike information criteria was selected as the final disease pattern. A score per subject was calculated by taking the inner product of the disease pattern and the subject’s image. To robustly test the generated scores, a leave-one-out cross-validation (LOOCV) was applied. A receiver operating characteristic (ROC) curve was plotted and the area under the curve (AUC) was calculated to assess the discriminating values of the pattern.

### Statistical analysis

Statistical analysis was performed using IBM SPSS Statistics version 23. All baseline data were quantitatively described. Pearson’s χ^2^ test, one-way ANOVA, Mann Whitney U or Kruskall Wallis tests were used to compare the demographic and clinical data between groups.

For the VOI based analysis, *BP*_ND_ values were compared between respectively the DRD and CD and DRD and healthy control group with a Mann Whitney U test. Correction for multiple comparisons (Bonferroni) was performed and the statistical significance threshold was set at a *p*-value < 0.004 (= 0.05/13 VOIs). An ANCOVA, after a square root transformation of the *BP*_*ND*_ values, was performed to correct for presence of psychiatric co-morbidity.

Voxel-based analysis was performed with SPM12 (Wellcome Trust Centre for Neuroimaging, UK) and a two-sample *t*-test was used to assess differences between DRD patients and CD patients, and DRD patients and healthy controls. T-maps data were interrogated at *p* = 0.005 (uncorrected) and only clusters with *p* < 0.05 corrected for family-wise error were considered significant.

Spearman’s rho test corrected for multiple testing (Benjamini–Hochberg) was used to perform a correlation analysis between the *BP*_ND_ in the VOIs and clinical variables in all participants. To assess differences in *BP*_ND_ between participants with a lifetime psychiatric diagnosis and those without, a Mann Whitney U test was performed. To determine whether the significant correlations that we found in the whole group were also present in the DRD group, above mentioned tests were also performed in the DRD group.

Finally, to check for possible confounders, a Spearman’s rho test was performed between dosage of levodopa, age, La/La genotype and *BP*_ND_.

### Ethics approval

This study was approved by the medical ethical committee of the UMCG (METc 034/14).

### Consent to participate

Written informed consent was obtained from all subjects according to the Declaration of Helsinki.

## Results

Demographic information and clinical characteristics can be found in Table 1. The median age of DRD patients was not significantly different compared to the control groups and no differences in smoking habit or La/La genotype were detected. Eight DRD patients were using levodopa (median daily dose of levodopa 150 mg, range 100–400 mg). One of the remaining two patients, who weren’t using levodopa, was an asymptomatic mutation carrier, and the other patient reported to have no symptoms but based on neurological examination did have mild dystonia (BFMDRS 9.5). No other dopaminergic drugs were used. Dystonia was well controlled in the DRD group with a median BFMDRS of 6.5 (max. score of 120), and a median CGI score of 2 (max. score of 7) which was significantly lower than in the CD group (median 4.5). No symptoms of Parkinsonism were observed in DRD group.

**Table 1:** Demographics, clinical characteristics and non-motor symptoms.

	DRD (n = 10)	CD (n = 14)	Controls (n = 12)	*p*-value
Age	46.5 (31–77)	55.0 (44–70)	53.5 (39–66)	0.40^a^
Gender (M/F)	2/8	2/12	3/9	0.87^b^
Smoking	1 (10%)	6 (43%)	2 (17%)	0.43^b^
La/La genotype	4 (40%)	3 (21%)	5 (42%)	0.48^b^
Injected dose of [^11^C]DASB (MBq)	389 (364–413)	387 (368–406)	371 (337–405)	0.53^c^
**Motor symptoms**				
Duration in years	40 (21–71)	9 (1–52)		0.00**^d^
BFMDRS	6.5 (0–17.5)	5.3 (2–12)		0.62^d^
CGI	2 (1–2)	4.5 (2–7)		0.00**^d^
**Non motor symptoms**				
BAI	6.5 (3–12)	8 (1–23)	3 (0–12)	0.02*^a^
BDI	5 (0–15)	11 (4–23)	2 (0–11)	0.01**^a^
ESS	10.5 (4–21)	12.5 (0–24)	4.5 (0–14)	0.01**^a^
FSS	31.5 (19–62)	36 (21–63)	22.5 (15–38)	0.00**^a^
PSQI	8.5 (4–13)	8 (1–16)	4.5 (1–12)	0.18^a^
Presence psychiatric diagnosis	6 (60%)	11 (79%)	4 (33%)	0.07^b^
Depression	4 (40%)	5 (36%)	2 (17%)	0.43^b^
Panic disorder	2 (20%)	3 (21%)	0 (0%)	0.23^b^
Agora phobia	5 (50%)	2 (14%)	0 (0%)	0.01**^b^
Social phobia	2 (20%)	2 (14%)	0 (0%)	0.35^b^
Generalized anxiety	3 (30%)	2 (14%)	0 (0%)	0.16^b^
OCD	1 (10%)	1 (7%)	0 (0%)	0.73^b^

Data presented as median (range), mean (95% confidence interval) or number (%).

*DRD* dopa-responsive dystonia, *CD* cervical dystonia, *BFMDRS* Burke Fahn Marsden Dystonia Rating Scale, *CGI* Clinical Global Impact Scale, *BAI* Beck Anxiety Index, *BDI* Beck Depression Index, *ESS* Excessive Sleepiness Scale, *FSS* Fatigue Severity Scale, *PSQI* Pittsburgh Sleep Quality Index, *OCD* obsessive compulsive disorder.

Kruskal wallis test^a^, fisher-freeman-halton exact test^b^ one-way ANOVA^c^ and Mann Whitney U tests^d^ were performed to compare the groups.

*p-value < 0.05, **p-value < 0.01.

Psychiatric co-morbidity was highly present in both dystonia groups (DRD 60%, CD 79%) compared to the controls (33%). The severity scores assessing severity of depression, anxiety, sleep problems and fatigue were also significantly different between the groups, with more severe non-motor symptoms in both dystonia groups (Table 1).

### VOI and voxel-based analysis

**Figure 1:**
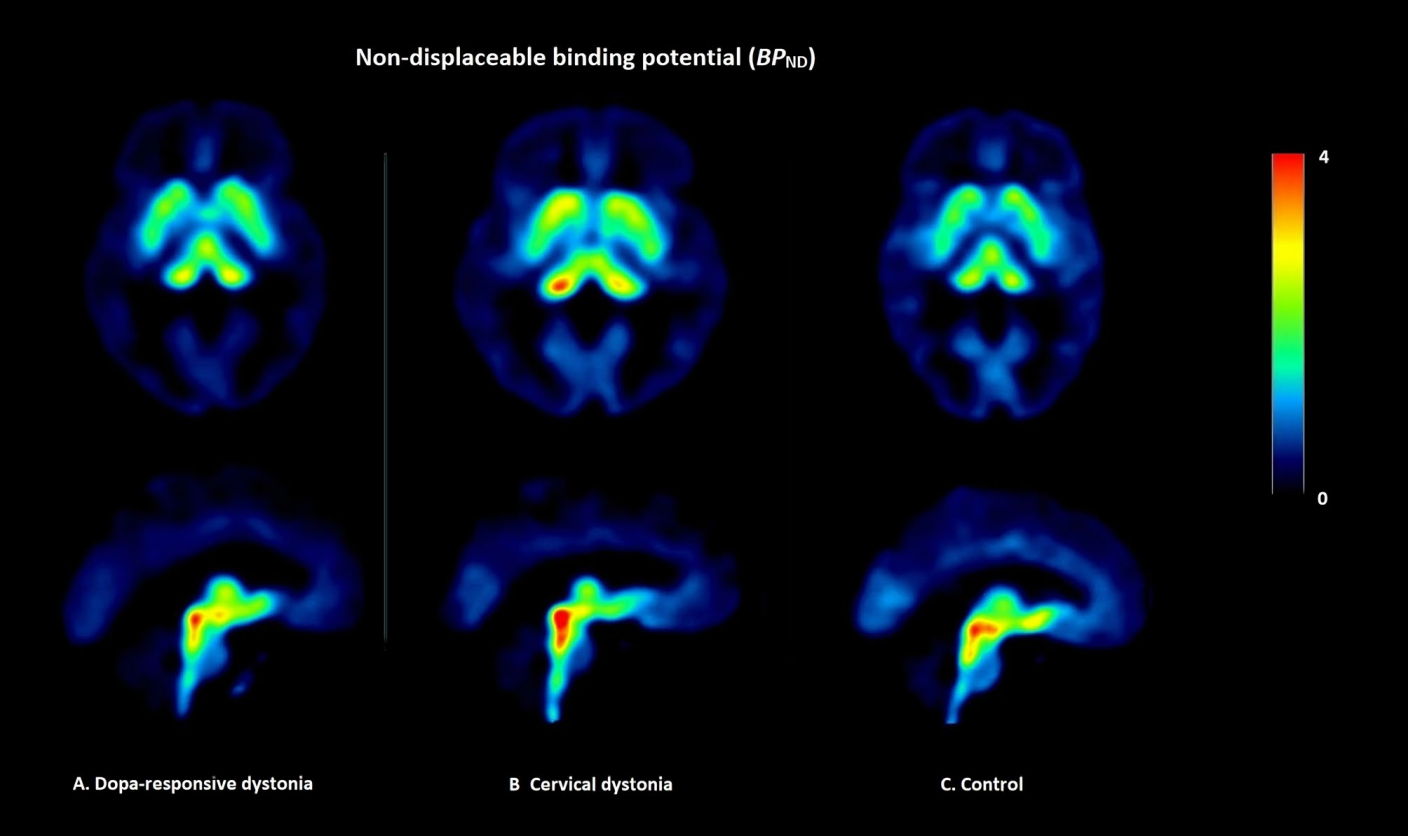
Non-displaceable binding potential (*BP*_*ND*_). Example of the non-displaceable binding potential of [^11^C]DASB in a dopa-responsive dystonia (DRD) patient, a cervical dystonia (CD) patient and a control participant without a movement disorder. The DRD patient had dystonia for 43 years, was diagnosed with a panic disorder and had sleep disturbances; the CD patient suffered from dystonia for 52 years, was diagnosed with depression and panic disorder and was easily fatigued, the healthy control had no history of a psychiatric disorder and or sleep disturbance.

Visual assessment of the [^11^C]DASB PET scans revealed high *BP*_ND_ in the dorsal midbrain, thalamus, and striatum in all groups (Fig. 1).

**Figure 2:**
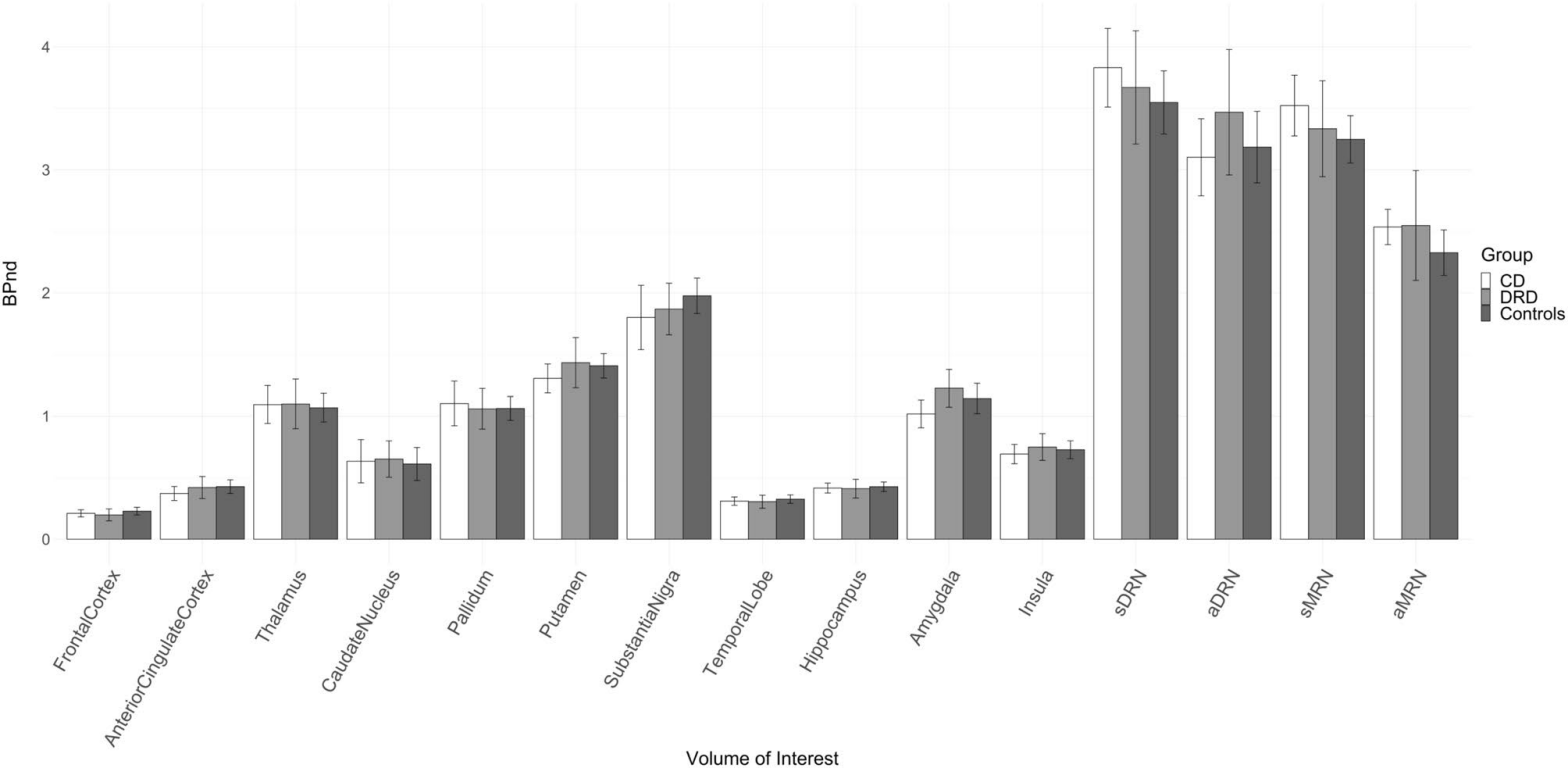
Non-displaceable binding potential (*BP*_ND_) per VOI. Data presented as mean with 95% confidence interval as error bar. No significant differences in any region between the groups were found. *CD* Cervical dystonia, *DRD* Dopa-responsive dystonia.

Further analysis showed no significant differences in the *BP*_ND_ of VOIs between the DRD and CD group, nor between the DRD and the healthy control group (Fig. 2). The mean volume of the VOIs was not statistically different between groups (Supplementary Table 1).

Accordingly, voxel-based analysis did not show any significant differences between the DRD and the two control groups.

### SSM/PCA analysis

**Figure 3:**
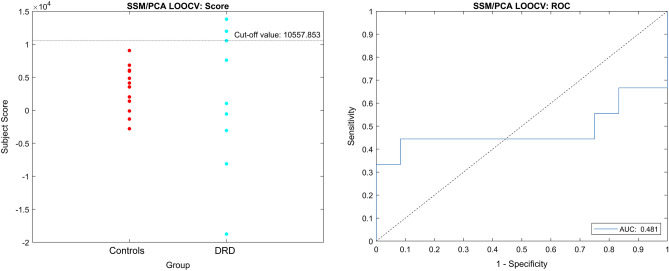
SSM/PCA analysis subject score and relative operating characteristic curve. Left: subject scores for the disease related covariate pattern after a leave-one-out cross-validation (LOOCV). Right: relative operating characteristic (ROC) curve for the disease-related covariate pattern after LOOCV with an area under the curve (AUC) of 0.5.

No disease-related covariance pattern could be created with SSM/PCA method that discriminates DRD from CD patients. We did create a disease-related covariance pattern comparing DRD patients to controls without a movement disorder. However, the subject scores were not significantly different between the groups (DRD vs. healthy controls: median 1042 vs. 3839, *p* = 0.77) and the AUC of the ROC curve was 0.48, indicating that the pattern did not effectively discriminate between the two groups (Fig. 3).

### Relationship (non-)motor symptoms and BP_ND_

No significant correlations between severity of the dystonia (CGI) and *BP*_ND_ in any of the VOIs was found in all participants nor in the DRD group (Supplementary Fig. 1).

**Figure 4:**
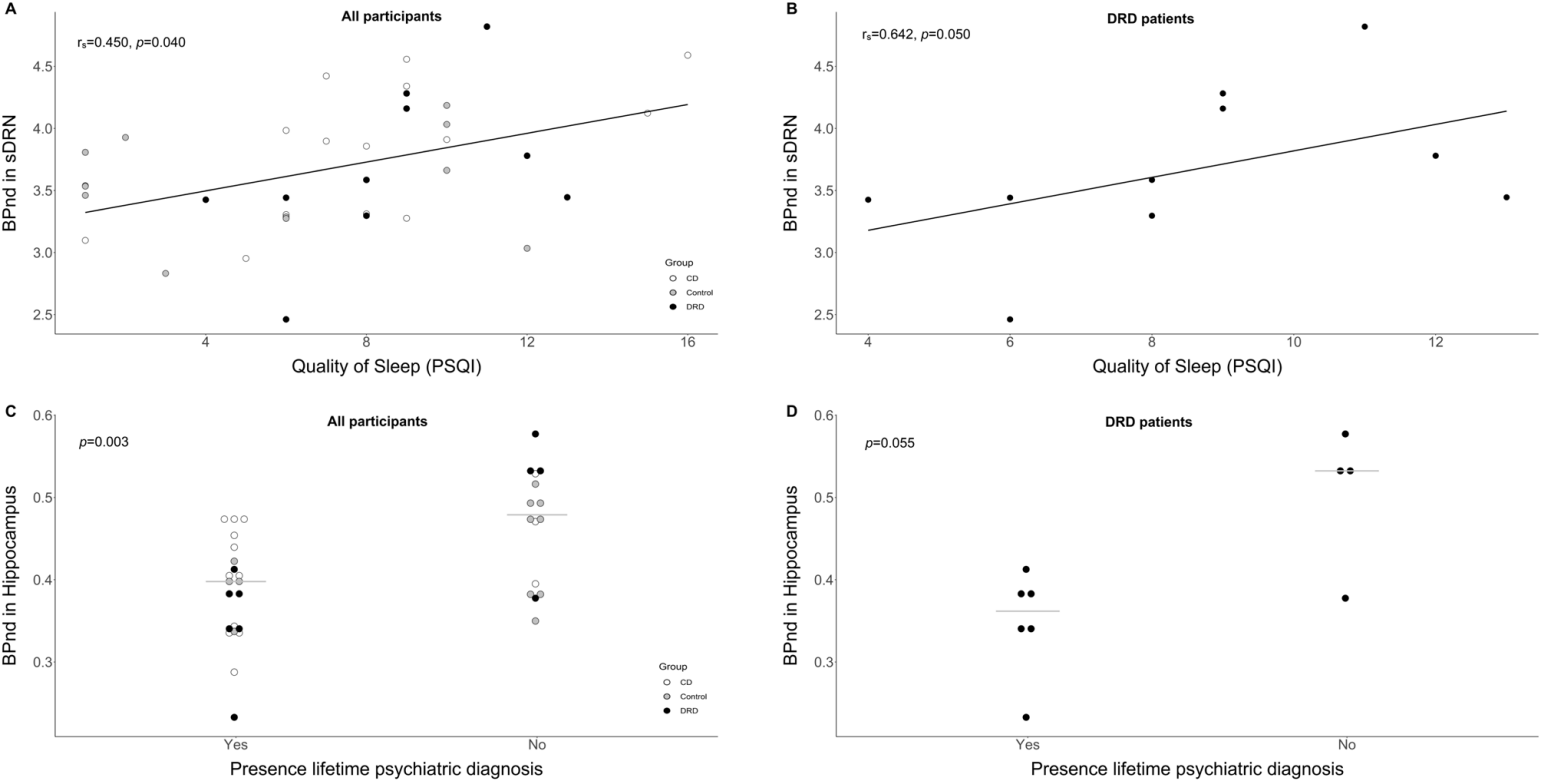
Associations between non-motor symptoms and non-displaceable binding potential (*BP*_*ND*_). (**A**) Correlation, calculated with Spearman’s rho test, between quality of sleep and *BP*_*ND*_ in dorsal raphe nucleus (sDRN) in all participants, p-value is corrected for multiple comparisons. (**B**) Correlation, calculated with Spearman’s rho test, between quality of sleep and* BP*_*ND*_ in sDRN in DRD patients, p-value is corrected for multiple comparisons. (**C**) Dotplot with median (line) of* BP*_*ND*_ in hippocampus and presence of a life time psychiatric diagnosis in all participants. (**D**) Dotplot with median (line) of* BP*_*ND*_ in hippocampus and presence of a life time psychiatric diagnosis in DRD patients. All results of the correlation analysis can be found in Supplementary Fig. S1.

In all participants a significant association was found between quality of sleep and *BP*_ND_ in the sDRN (r_s_ = 0.45, *p* = 0.04), indicating a higher *BP*_ND_ in participants with a worse quality of sleep. This positive association was also present in the DRD patients, this almost reached statistical significance after correction for multiple comparisons (r_s_ = 0.64, *p* = 0.05) (Fig. 4). In DRD patients, a negative association between fatigue and the *BP*_ND_ in the sMRN (r_s_ =  − 0.66, *p* = 0.05) was also found, indicating a lower *BP*_ND_ in patients with more fatigue. This finding was not present in the whole group.

Participants who were at least once in their life diagnosed with a psychiatric disorder had a lower *BP*_ND_ in the hippocampus (median 0.40 vs. 0.48*, p* = 0.00). In DRD patients this finding was also present, but did not reach statistical significance (median 0.36 vs. 0.53, *p* = 0.06).

### Post hoc analysis

A post hoc analysis in which we corrected for the presence of a psychiatric disorder showed a significant difference in *BP*_*ND*_ in the hippocampus between DRD patients and respectively CD patients and healthy controls (uncorrected *p*-value = 0.004, Supplementary Table 2).

### Confounders

In the DRD group no correlations between dosage of levodopa and *BP*_ND_ in any of the VOIs was found, and, visually, no differences in *BP*_ND_ were observed in the eight patients who were using levodopa versus the two who were not. No correlations between age and *BP*_ND_ in any of the VOIs were found in the whole group. The La/La genotype was associated with a lower *BP*_ND_ (r_s_ = 0.48, *p* = 0.03) in the globus pallidus.

## Discussion

This is the first in vivo study assessing the serotonergic brain metabolism in patients with GTP cyclohydrolase deficient DRD using [^11^C]DASB PET scans. Both the univariate- as well as the network analysis did not show any differences in SERT binding between DRD patients compared to CD patients or healthy controls. However, the highly prevalent non-motor symptoms did have an association with SERT binding; sleep disturbances and psychiatric co-morbidity were correlated with respectively the *BP*_ND_ in the raphe nuclei and hippocampus.

The deficiency of the enzyme GTP-cyclohydrolase 1 in patients with DRD leads to an impaired synthesis of BH4, resulting in a decreased synthesis of dopamine known to cause motor symptoms, while a decreased synthesis of serotonin hypothetically can be linked to the non-motor symptoms. However, we did not observe any differences in brain SERT binding in patients with DRD, suggesting that the number of SERT or their binding capacity was not different. Our finding is in line with a post-mortem case report that did not show altered levels of serotonin markers, including SERT protein in the striatum of a DRD patient^22^. On the other hand, low levels of 5-HIAA, one of the main downstream metabolites of serotonin, are reported in the CSF of some untreated DRD patients, which do suggest an altered serotonergic metabolism^3,7^. One important consideration is that with [^11^C]DASB PET scans we measured SERT, an important regulator of the synaptic concentration of serotonin, but we did not directly measure serotonin concentrations.

Another explanation for our negative findings could be that alterations in the serotonergic metabolism in DRD are too subtle to be detected with a single PET scan assessing only the binding to SERT. As we know that other neurotransmitters, such as dopamine and acetylcholine, are also involved in dystonia, it is very likely that the non-motor symptoms, in a similar way, can be the result of an imbalance between multiple neurotransmitters, rather than a disruption in the pathway of only serotonin. Other neurotransmitters such as dopamine, noradrenaline and acetylcholine are extensively studied with regard to psychiatric disorders and sleep disorders^23–25^ and are likely involved in non-motor symptoms in dystonia as well. Further studies assessing not only the serotonergic system, but combined with other neurotransmitter systems are necessary.

Assessing all participants we did, however, find an association between psychiatric co-morbidity and SERT binding in the hippocampus, indicating that the serotonergic system is involved in psychiatric disorders. A subsequently performed post-hoc analysis to correct for psychiatry in the VOI-based analysis revealed a significant difference in SERT binding in the hippocampus between DRD patients and the two control groups. This suggests that there might be an altered serotonergic system related to the motor symptoms in DRD patients, overshadowed by the high prevalence of psychiatric disorders and corresponding disturbances in the serotonergic system. This is a difficult concept as the psychiatric symptoms are currently considered part of the phenotype in DRD patients. Further studies in a larger group of DRD patients is required to further study the relationship of DRD patients with and without psychiatric symptoms with the serotonergic system.

Furthermore, the correlation analysis in all participants showed that a worse quality of sleep was associated with higher SERT binding in the DRN. This was also observed in the DRD group, though the correlation did not reach statistical significance after correction for multiple comparisons. In the past, several studies showed evidence of the complex interaction between the raphe nuclei and sleep^26^. Serotonin has a dual function in sleep and waking and this is now believed to depend, among other things, not only on the brain region and serotonin receptor type involved, but also on the interaction with other neurotransmitter systems^27^. This might explain the contrasting finding of an association between fatigue and a lower *BP*_ND_ in the median raphe nucleus in DRD patients. Again, the correlation was not statistically significant after correction for multiple comparisons (*p* = 0.05), probably due to the small sample size. Comparable results were found in two [^11^C]DASB studies in Parkinson’s disease, where patients with sleep dysfunction or fatigue had a lower *BP*_ND_ in several brain areas including the raphe nuclei as compared to patients without sleep problems^28,29^. Further studies assessing the role of serotonin in sleep and fatigue in dystonia patients are warranted to validate the findings of our study. Our results support that the serotonergic system is associated with both sleep and psychiatric disorders, but that this is not specific for DRD.

This study has several limitations that should be taken into consideration. The levodopa therapy that our patients were taking might have influenced our results. However, it was shown that levodopa therapy is able to reduce levels of serotonin and 5-HIAA in the brain even more^30^, so one would expect to find more differences in SERT binding. Furthermore, we did not find a correlation between dosage of levodopa and *BP*_ND_ in any of the VOIs in the DRD group. None of the participants were taking serotonergic medication during the study, however, one DRD patient used paroxetine 7 years prior to the scan, this might have had some influence on our results. Next, the number of participants was relatively small which is an inevitable reality when one investigates a rare disorder such as GTP cyclohydrolase deficient DRD, but this may account for our negative findings.

To conclude, no differences in SERT binding in DRD patients were found, indicating that either there is no altered serotonergic metabolism, or differences are too subtle to be detected with [^11^C]DASB PET scans alone. Associations between sleep problems, psychiatric diagnosis, and SERT binding were found in the whole group and were not specific for DRD. However, when corrected for psychiatric co-morbidity a significant difference in SERT binding in the hippocampus was found between the DRD patients and controls. This suggests an altered serotonergic metabolism in DRD, masked by the high prevalence of psychiatric disorders. Further research investigating psychiatric co-morbidity and multiple neurotransmitters, like dopamine, norepinephrine or acetylcholine together with serotonin, are necessary to shed light on the pathophysiology of the highly present non-motor symptoms in DRD.

## Supplementary Information

Supplementary Information.

## Data Availability

Raw data are available upon reasonable request, after signing a data use agreement of the UMCG.

## Abbreviations

5-HIAA: 5-Hydroxyindoleacetic acid
5-HTTLPR: Serotonin reuptake transporter-linked polymorphic region
[^11^C]DASB: [^11^C]-3-amino-4-(2-dimethylaminomethylphenylsulfanyl)benzonitrile
BAI: Beck Anxiety Index
BDI: Beck Depression Inventory
BFMDRS: Burke Fahn Marsden Dystonia Rating Scale
BH4: Tetrahydrobiopterin
CD: Cervical dystonia
CGI: Clinical Global Impression Scale
DRD: Dopa-responsive dystonia
DRN: Dorsal raphe nucleus
ESS: Epworth Sleepiness Scale
FSS: Fatigue Severity Scale
MINI-PLUS: Mini International Neuropsychiatric Interview—PLUS
MRN: Median raphe nucleus
PSQI: Pittsburgh Sleep Quality Index
SSM/PCA: Scaled subprofile model based on principal component analysis
SERT: Serotonin reuptake transporter
