# Supplementary material for: Serotonergic system in vivo with [11C]DASB PET scans in GTP-cyclohydrolase deficient dopa-responsive dystonia patients
Source: Sci Rep. 2022 Apr 15;12:6292. doi: 10.1038/s41598-022-10067-5 (PMC9012759; doi:10.1038/s41598-022-10067-5)
Supplement: Supplementary file 1 — Supplementary Information. [file 41598_2022_10067_MOESM1_ESM.pdf]

Supplementary table 1. Mean volume of Volumes of Interest (VOI)

	DRD	CD	Controls	*p-value*
**Frontal Cortex**	155 (145-165)	149 (144-155)	156 (146-167)	0.42
**Anterior Cingulate Cortex**	10.6 (9.9-11.3)	10.2 (9.7-10.7)	10.9 (10.2-11.6)	0.16
**Thalamus**	13.8 (12.7-14.9)	14.3 (9.7-10.7)	14.4 (10.2-11.6)	0.51
**Caudate Nucleus**	8.3 (7.6-9.1)	8.7 (8.3-9.2)	8.7 (8.0-9.4)	0.54
**Pallidum**	2.4 (2.3-2.5)	2.5 (2.4-2.6)	2.5 (2.4-2.7)	0.49
**Putamen**	8.9 (8.4-9.4)	9.3 (9.0-9.7)	9.5 (8.9-10.1)	0.26
**Substantia Nigra**	0.6 (0.6-0.6)	0.6 (0.6-0.7)	0.6 (0.6-0.7)	0.36
**Temporal Lobe**	132 (124-140)	128 (124-133)	136 (127-145)	0.23
**Hippocampus**	4.2 (4.0-4.4)	4.3 (4.2-4.5)	4.4 (4.1-4.7)	0.30
**Amygdala**	2.7 (2.5-2.8)	2.8 (2.7-2.9)	2.8 (2.6-3.0)	0.32
**Insula**	17.1 (15.9-18.3)	16.5 (15.7-17.2)	17.4 (16.1-18.8)	0.35
**sDRN**	0.2 (0.2-0.3)	0.2 (0.2-0.2)	0.2 (0.2-0.3)	0.14
**sMRN**	0.1 (0.0-0.1)	0.1 (0.1-0.1)	0.1 (0.1-0.1)	0.72
**aDRN**	0.1 (0.1-0.1)	0.1 (0.1-0.1)	0.1 (0.1-0.1)	0.30
**aMRN**	0.1 (0.1-0.1)	0.1 (0.1-0.1)	0.1 (0.1-0.1)	0.82

Data are presented in ccm (95% CI). One way ANOVA was performed to calculate p-values. DRD: dopa-responsive dystonia, CD: cervical dystonia, sDRN: subject-based dorsal raphe nucleus, sMRN: subject-based median raphe nucleus, aDRN: atlas-based dorsal raphe nucleus, aMRN: atlas-based median raphe nucleus.

Supplementary table 2. Differences in BP_{ND} between the groups, corrected for psychiatric co-morbidity

	DRD	CD	p-*value*[a]	Controls	p-*value*[b]
**Frontal Cortex**	0.20 (0.07)	0.21 (0.05)	*0.902*	0.23 (0.05)	*0.655*
**Anterior Cingulate Cortex**	0.42 (0.12)	3.72 (0.40)	*0.577*	0.43 (0.09)	*0.760*
**Thalamus**	1.10 (0.28)	1.10 (0.27)	*0.295*	1.07 (0.18)	*0.295*
**Caudate Nucleus**	0.65 (0.21)	0.63 (0.30)	*0.751*	0.62 (0.21)	*0.081*
**Pallidum**	1.06 (0.23)	1.10 (1.03)	*0.111*	1.06 (0.15)	*0.596*
**Putamen**	1.43 (0.28)	1.30 (0.20)	*0.611*	1.41 (0.16)	*0.843*
**Substantia Nigra**	1.87 (0.29)	1.80 (0.45)	*0.259*	1.98 (0.23)	*0.110*
**Temporal Lobe**	0.31 (0.07)	0.31 (0.06)	*0.395*	0.33 (0.05)	*0.460*
**Hippocampus**	0.41 (0.11)	0.42 (0.07)	*0.004**	0.43 (0.06)	*0.004**
**Amygdala**	1.23 (0.21)	1.02 (0.20)	*0.234*	1.14 (0.19)	*0.025*
**Insula**	0.75 (0.15)	0.69 (0.14)	*0.667*	0.73 (0.11)	*0.629*
**sDRN**	3.67 (0.64)	3.83 (0.55)	*0.029*	3.55 (0.41)	*0.007*
**sMRN**	3.33 (0.54)	3.52 (0.43)	*0.027*	3.25 (0.31)	*0.221*
**aDRN**	3.47 (0.71)	3.10 (0.54)	*0.018*	3.18 (0.46)	*0.051*
**aMRN**	2.55 (0.62)	2.53 (0.25)	*0.401*	2.33 (0.29)	*0.474*

Data is shown as mean (SD). ANCOVA with 'presence of a psychiatric disorder' as a covariate was used to compute p-values. Original BP_{ND} values were not normally distributed, and due to the small sample sizes a square-root transformation was applied before the analysis was performed. The first p-value[a] is the result of comparison between DRD and CD patients, the second p-*value*[b] *depicts the comparison between DRD patients and healthy controls.* P-*values are given uncorrected for multiple comparisons.* *p-*value considered statistically significant with a Bonferroni correction for multiple comparisons (p*-value <0.004 (=0.05/13 VOIs)).

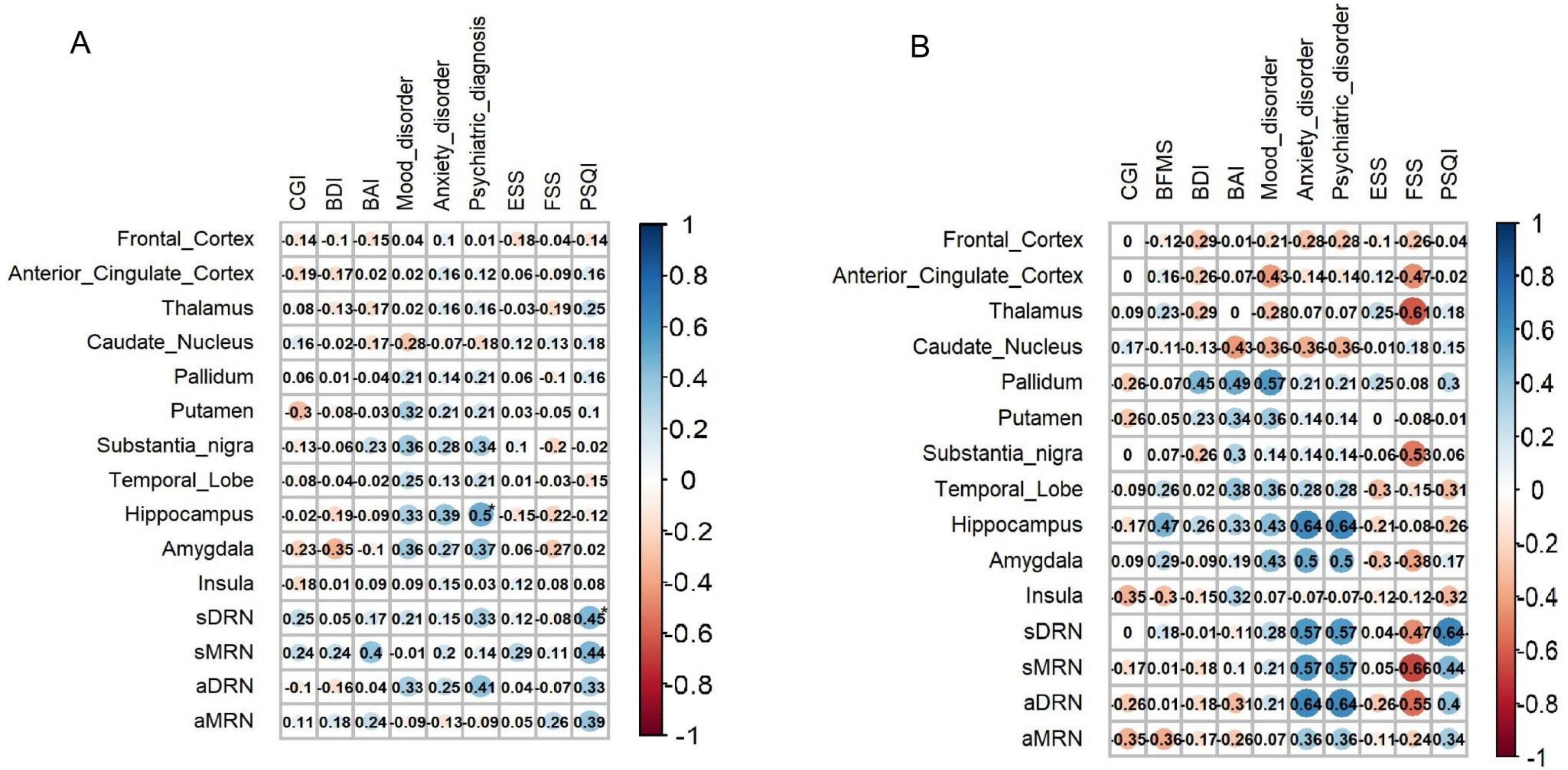

Supplementary figure 1. Correlation coefficients calculated with Spearman's rho test in all participants (A) and DRD patients (B). * *p*<0.05 after Benjamini-Hochberg correction for multiple comparisons.
